# Fecal propionate is a signature of insulin resistance in polycystic ovary syndrome

**DOI:** 10.3389/fcimb.2024.1394873

**Published:** 2025-01-13

**Authors:** Sitong Dong, Xinrui Yao, Jiao Jiao, Bei Lin, Fujie Yan, Xiuxia Wang

**Affiliations:** ^1^ Center of Reproductive Medicine, Shengjing Hospital of China Medical University, Shenyang, China; ^2^ Shenyang Reproductive Health Clinical Medicine Research Center, Shenyang, China; ^3^ Department of Gynecology and Obstetrics, Shengjing Hospital of China Medical University, Shenyang, China; ^4^ Key Laboratory of Maternal-Fetal Medicine of Liaoning Province, Shenyang, China; ^5^ Key Laboratory of Obstetrics and Gynecology of Higher Education of Liaoning Province, Shenyang, China; ^6^ Department of Food Science and Nutrition, College of Biosystems Engineering and Food Science, Zhejiang University, Hangzhou, China; ^7^ Key Laboratory of Reproductive and Genetic Medicine, China Medical University, National Health Commission, Shenyang, China

**Keywords:** propionate, polycystic ovary syndrome, insulin resistance, *Prevotella copri*, *Megamonas funiformis*

## Abstract

**Objective:**

To investigate the roles of fecal short-chain fatty acids (SCFAs) in polycystic ovary syndrome (PCOS).

**Methods:**

The levels of SCFAs (acetate, propionate, and butyrate) in 83 patients with PCOS and 63 controls were measured, and their relationships with various metabolic parameters were analyzed. Intestinal microbiome analysis was conducted to identify relevant bacteria. The study took place at the Center for Reproductive Medicine at Shengjing Hospital of China Medical University in Shenyang, from 5 February to 23 May 2023. Logistic regression analyses were used to investigate the relationships between SCFAs, PCOS, and PCOS-related insulin resistance (IR). Differences in bacterial populations between women with PCOS-IR and those with PCOS-non-insulin resistance (NIR) were identified using linear discriminant analysis effect Size (LEfSe). The relationships between bacteria and fecal propionate levels were explored through linear regression analyses. The potential of fecal propionate and microbial profiles as biomarkers for insulin resistance in PCOS patients was assessed using receiver operating characteristic (ROC) curve analysis.

**Results:**

Higher fecal propionate levels were observed in patients with PCOS compared to controls (*p* = 0.042) and in PCOS-IR compared to PCOS-NIR (*p* = 0.009). There was no significant difference in fecal propionate levels between the IR and NIR subgroups of women in the control group (*p* > 0.05). Additionally, higher fecal propionate levels were associated with IR in PCOS (*p* = 0.039; OR, 1.115; 95% CI, 1.006–1.237). The abundance of *Prevotella copri* and *Megamonas funiformis* was higher in PCOS-IR women compared to PCOS-NIR women (LDA score > 3) and correlated with fecal propionate levels (adjusted *R*² = 0.145, *p* < 0.001). The area under the curve (AUC) for propionate and the combined presence of *P. copri* and *M. funiformis* in predicting PCOS was 78.0%, with a sensitivity of 78.5% and a specificity of 72.4%. Pathways related to carbohydrate metabolism were significantly enriched in the microbiota of the PCOS-IR population but not in the control IR group.

**Conclusions:**

Higher fecal propionate levels correlate with PCOS-related insulin resistance. *P. copri* and *M. funiformis* might be key functional bacteria. Therefore, the combination of propionate levels and the abundance of these two bacteria may serve as a potential biomarker for insulin resistance in PCOS patients. Regulation of the intestinal microbiome might be beneficial for the metabolic health of women with PCOS.

## Introduction

Polycystic ovarian syndrome (PCOS) is a complex reproductive and endocrine disease with a high prevalence, affecting 5%–20% of women of reproductive age (Azziz, 2018). It is characterized by ovulatory dysfunction, hyperandrogenism, and polycystic ovarian morphology. However, the exact etiology and pathogenesis of PCOS remain unknown. Women with PCOS commonly exhibit multiple metabolic complications, such as obesity, dyslipidemia, insulin resistance (IR), and compensatory hyperinsulinemia (Rosenfield and Ehrmann, 2016). They also have an increased risk of developing type 2 diabetes mellitus (T2DM), infertility, obstetrical complications, endometrial cancer, and mood disorders. Obesity and hyperinsulinemia are significant contributors to the development of PCOS (Dobbie et al., 2023). Adipose tissue has been shown to induce ovulation disorders (Zhou et al., 2021). Additionally, changes in diet and lifestyle can help reduce PCOS-related metabolic abnormalities (Gu et al., 2022b). IR appears to increase androgen production by acting on the pituitary, ovaries, and liver (Barbieri et al., 1986); inhibit sex hormone-binding globulin (SHBG) and increase androgen levels (Plymate et al., 1988); and interfere with follicular development, leading to anovulation (Poretsky et al., 1999). IR also promotes the development of metabolic syndrome in PCOS patients and independently increases the risk of cardiovascular dysfunction (Ovalle and Azziz, 2002). Compared to other insulin-resistant states, the molecular mechanisms underlying IR in PCOS appear to be distinct.

The microbiota colonized in the human body engages in complex activities (Gu et al., 2022a; Zhou et al., 2022), producing various metabolites such as lipopolysaccharides, peptidoglycans, trimethylamine, secondary bile acids, and short-chain fatty acids (SCFAs). These metabolites are involved in crucial physiological processes, such as aiding digestion and regulating immunity, as well as pathological processes like inflammation and metabolic abnormalities (Nicholson et al., 2012). Intestinal microbiota dysbiosis has been observed in women with PCOS, and its metabolites have been shown to play a vital role in the pathogenesis of this condition (Qi et al., 2019; Gu et al., 2022a). SCFAs are produced locally in the colon through microbial fermentation of undigested dietary fibers and resistant starch. Major SCFAs are acetate, propionate, and butyrate (Cook and Sellin, 1998). They have essential roles in maintaining intestinal homeostasis, regulating glucose and energy metabolism, and modulating immune responses (Koh et al., 2016). Studies have shown that fecal SCFA levels increase in populations with obesity and non-alcoholic fatty liver disease (NAFLD) (Samuel et al., 2008; Rau et al., 2018). Additionally, there are suggested causal relationships between butyrate and improved oral glucose test results, as well as propionate and an increased risk of T2DM (Sanna et al., 2019). These metabolic abnormalities have a higher incidence in PCOS (Azziz, 2018; Kumarendran et al., 2018). Changes in fecal SCFA levels have been observed in the PCOS population and have been correlated with sex hormone regulation in PCOS patients following probiotic use (Zhang et al., 2019; Li et al., 2022). However, the relationship between fecal SCFAs and metabolic abnormalities in PCOS warrants further investigation.

This study aimed to evaluate fecal SCFA levels in women with PCOS compared to healthy women, investigating the relationship between fecal SCFAs and both PCOS and PCOS-related metabolic status. Additionally, we identified specific gut microbiota species associated with changes in propionate levels and developed a combined classifier to distinguish insulin-resistant (IR) from non-insulin-resistant (NIR) patients with PCOS.

## Methods

### Subject recruitment

**Figure 1:**
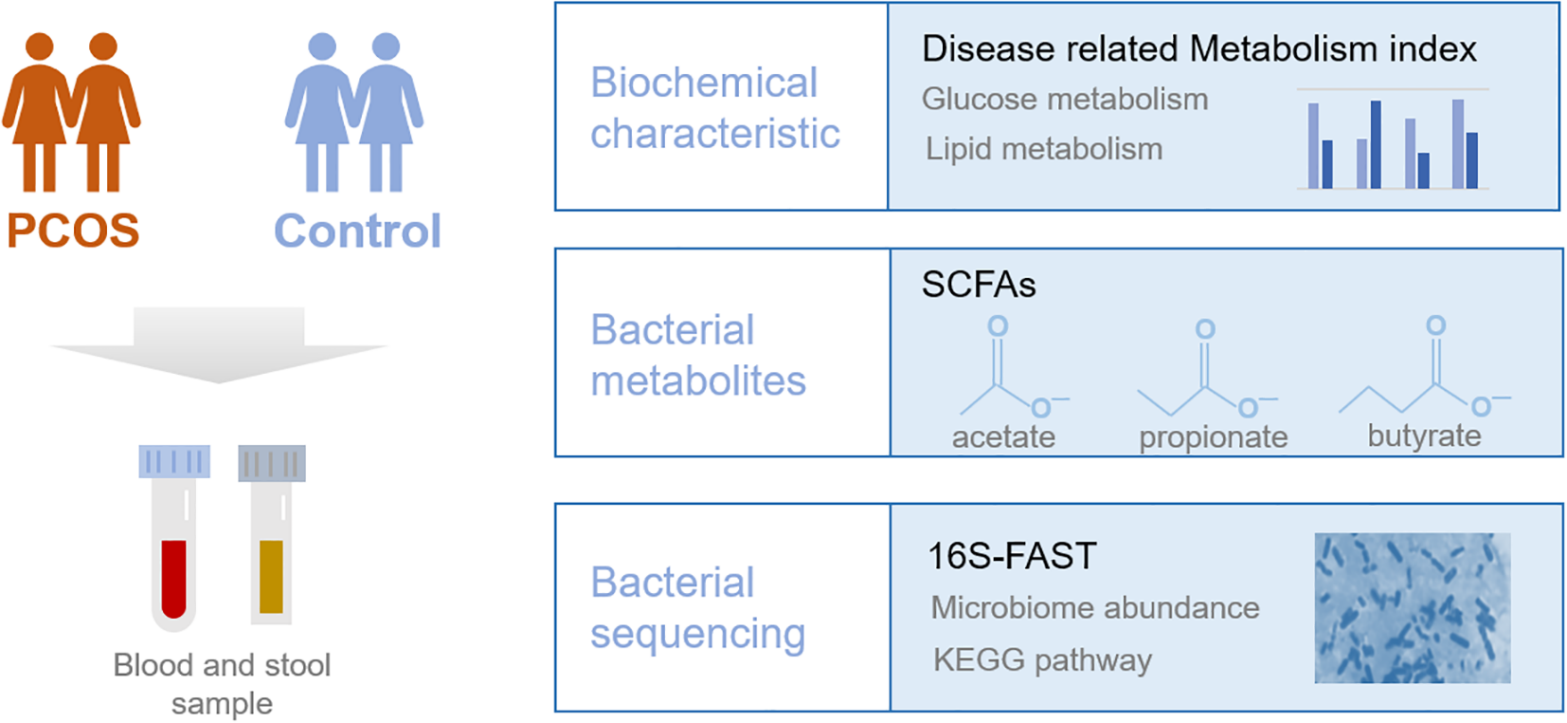
Diagram of the study design.

This retrospective case–control study recruited women from the Center for Reproductive Medicine at Shengjing Hospital of China Medical University between 5 February and 23 May 2022. According to the Rotterdam criteria (Revised 2003 consensus on diagnostic criteria and long-term health risks related to polycystic ovary syndrome (PCOS), 2004), women were diagnosed with PCOS if they presented with any two of the following three conditions: oligo- or anovulatory cycles, clinical or biochemical signs of hyperandrogenism after excluding other etiologies, or polycystic ovary manifestations. The control group consisted of women without PCOS who were clinically infertile due to fallopian tube issues or male factors. The exclusion criteria were consistent with our previous publications (Dong et al., 2021): patients who were smokers or alcoholics, pregnant, breastfeeding, on antibiotics or hormone medications within the past 6 months, or had other diseases or were on medications within the past 6 months that were known to influence the composition of the intestinal microbiome. Other reasons considered unsuitable for this study also led to exclusion. Ultimately, 146 women (83 patients with PCOS and 63 non-PCOS patients) participated in this study and provided written informed consent at enrolment. All procedures adhered to the Declaration of Helsinki and were approved by the Ethical Review Board of China Medical University (Approval Number 2015PS108K). The study design is illustrated in **Figure 1**.

### Collection of general information and biochemical sampling and measurement

Age and BMI were recorded from an electronic medical record database. On the day of sample collection, we conducted a brief survey on the participants’ dietary habits over the past week, categorized into the following three types: 1) vegetarian-based diet: primarily plant-based foods, avoiding or completely excluding animal-derived foods, including dairy and eggs; 2) meat-based diet: strong preference for animal-derived foods, with minimal intake of plant-based foods; and 3) meat and vegetarian diet: a balanced preference for both animal-derived and plant-based foods in daily meals. Venous blood samples were collected in the morning after overnight fasting on the second to fourth day of a spontaneous menstrual cycle or after progestin withdrawal bleeding. Total cholesterol (CHOL), triglycerides (TG), high-density lipoprotein cholesterol (HDL-C), low-density lipoprotein cholesterol (LDL-C), small dense low-density lipoprotein cholesterol (sd-LDL), fasting plasma glucose (FPG), and sex hormone-binding globulin (SHBG) were measured by enzyme-linked immunosorbent assay. Follicle-stimulating hormone (FSH), luteinizing hormone (LH), estradiol (E2), total testosterone (TT), prolactin (PRL), progestin (Prog), and fasting plasma insulin (FINS) were measured by chemiluminescence immunoassay. Anti-Müllerian hormone (AMH) was measured by enzyme immunoassay. The free androgen index (FAI) was calculated as 100 × TT (ng/mL) × 3.467/SHBG (nmol/L), and the Homeostatic Model Assessment for Insulin Resistance (HOMA-IR) was calculated as FPG (mM) × FINS (mIU/L)/22.5.

### 16S full-length assembly sequencing and microbiota analysis and determination of fecal SCFA concentrations

Fecal samples were collected in the morning during the non-menstrual period. Participants were instructed to collect fecal samples into fecal DNA storage tubes (CW2654, CwBiotech, Beijing, China). According to the kit’s instructions, approximately 1 g of stool sample should be collected and quickly placed into the preservation tube. The tube contains a preservation solution composed of NaCl, NaOH, EDTA, and purified water. This solution enables the DNA in the stool to be preserved at room temperature, effectively preventing microbial changes caused by temperature fluctuations and oxidation. The samples were stored at room temperature and then sent to the laboratory. Bacterial DNA was extracted using a DNA extraction kit (Qiagen Fecal DNA Extraction Kit, Qiagen, Hilden, Germany). Quantitative and qualitative analyses, as well as quality control of the extracted DNA, were performed. Sequencing was conducted following the methods described in our previous study (Dong et al., 2021). Specifically, 5′-AGRGTTYGATYMTGGCTCAG-3′ was used as the forward primer and 5′-RGYTACCTTGTTACGACTT-3′ as the reverse primer for the 16S full-length sequence. Default base quality filtering was applied using fastp. Sequences corresponding to unique tag pairs from the read-tag library were assembled to generate full-length 16S rRNA gene sequences using SPAdes v3.13.1 with default parameters. Full-length 16S rRNA gene sequences (≥1,200 bp) were then grouped into operational taxonomic units (OTUs) with 99% sequence similarity using VSEARCH in QIIME 2. OTUs meeting the criteria of –p-min-frequency 2 were retained using filter-features in QIIME 2. Chimeric feature sequences were detected using the vsearch uchime_denovo method, and OTUs were annotated with the SILVA_132_SSURef_Nr99 (Yilmaz et al., 2014) reference database using mothur v1.42.0. Only bacteria with an abundance greater than 0.01% in at least 10 samples were retained for differential bacterial analysis. Fecal SCFA concentrations were determined using the same method presented in our previous study (Yao et al., 2023).

### Grouping of participants

Considering the impact of propionate on metabolism and the metabolic phenotype of PCOS, participants were divided into different groups based on the presence or absence of several metabolic disorders, and fecal SCFA levels were compared.

Being overweight or obese is defined as having a BMI of ≥23 kg/m^2^, according to the World Health Organization’s definition for Asians (Appropriate body-mass index for Asian populations and its implications for policy and intervention strategies, 2004). To explore the relationship between fecal SCFA levels and BMI in the context of PCOS, both the PCOS and control groups were divided into normal weight and overweight or obese subgroups (BMI < 23 and ≥23 kg/m^2^, respectively).

IR was diagnosed based on the HOMA-IR formula, with a critical point set at greater than 2.5 (Einhorn et al., 2003). Therefore, to investigate the relationship between fecal SCFA levels and IR in the context of PCOS, each subject within the PCOS and control groups was classified as either non-insulin-resistant or insulin-resistant (HOMA-IR < 2.5 or ≥2.5, respectively).

Dyslipidemia was diagnosed if any one of the following conditions was met: TC ≥6.2 mmol/L, TG ≥2.3 mmol/L, LDL-C ≥4.1 mmol/L, or HDL-C <1.0 mmol/L (Kopin and Lowenstein, 2017). Based on these criteria, patients with PCOS and control women were subdivided into dyslipidemia and normolipidemia groups, respectively.

Metabolic syndrome (MS) was defined according to the criteria of the American Association of Clinical Endocrinologists/American College of Endocrinology (Einhorn et al., 2003), requiring any three of the following five conditions: BMI ≥25 kg/m^2^, TG ≥1.70 mmol/L, HDL-C <1.29 mmol/L, blood pressure ≥130/85 mmHg, 2 h postprandial plasma glucose >7.8 mmol/L, or FPG between 6.1 and 7.0 mmol/L. Additional risk factors included T2DM, family history of hypertension or cardiovascular diseases, PCOS, sedentary lifestyle, older age, and ethnicity with a high risk of T2DM or cardiovascular disease. The PCOS and control groups were subsequently stratified by the presence or absence of metabolic syndrome.

### Statistical analyses

A Kolmogorov–Smirnov assessment was performed to assess the normality of continuous variable distributions. For normally distributed variables, the mean and standard deviation were reported, whereas the median and interquartile range were used for variables with non-normal distributions. To compare data between the two groups, Student’s *t*-test was used for normally distributed data, while the Mann–Whitney *U* test was applied to non-normally distributed variables.

Categorical data were analyzed using Fisher’s exact test and the chi-square test. The correlation between fecal propionate concentration and metabolism parameters was assessed using Spearman’s correlation coefficient. The relationship between fecal propionate levels and FINS was further evaluated with univariate and multivariate linear regressions. Univariate logistic regression analysis was employed to determine if fecal propionate concentration was associated with IR in PCOS. After adjusting for other related indexes, multivariate logistic regression analysis estimated the odds ratio (OR) and 95% confidence interval (CI) of fecal propionate’s association with IR in PCOS. Receiver operating characteristic (ROC) curves were used to evaluate the diagnostic value of propionate and intestinal bacterial abundance for IR in PCOS women. For the combined indicators, a multivariable logistic regression model was constructed using glm. Data analyses were performed using SPSS version 23.0, and the results were visualized using GraphPad Prism 8 and R 4.3.1.

Alpha diversity was assessed using the Shannon index based on the OTU table with QIIME 2. Differences between groups were calculated by a Mann–Whitney *U* test using the “scipy 1.3.1” package in python 3.6. Beta diversity was analyzed using the “vegan 2.5-3” package in R3.6.1. Partial least squares-discriminate analysis (PLS-DA) was performed using Bray–Curtis distances to describe microbial composition and abundance. Adonis was employed to compute *p*-values to assess significant differences between the two groups. The identity threshold for accurately representing species was approximately 99% for full-length sequences (Edgar, 2018). The 16S rRNA sequences were obtained in their entirety and grouped into OTUs based on a 99% sequence similarity. Functional characteristics were predicted with PICRUSt2 version 2.5.2 (Douglas et al., 2020). Box plots were used to display the percentage of bacterial community abundance at the genus level. Differential functions were analyzed using linear discriminant analysis effect size (LEfSe) version 1.0 with default parameters. The correlation between bacterial species and metabolic indicators was conducted with the “psych v1.8.4” package in R3.6.1, using Spearman’s method. Heatmaps were generated using the “heatmap 1.0.12” R package. The beta coefficient of bacterial abundance changes relative to fecal propionate levels and multivariate linear regression analyses between predictors and fecal propionate levels were visualized using “ggplot2.”.

## Results

### Patients with PCOS presented with higher fecal propionate levels

**Figure 2:**
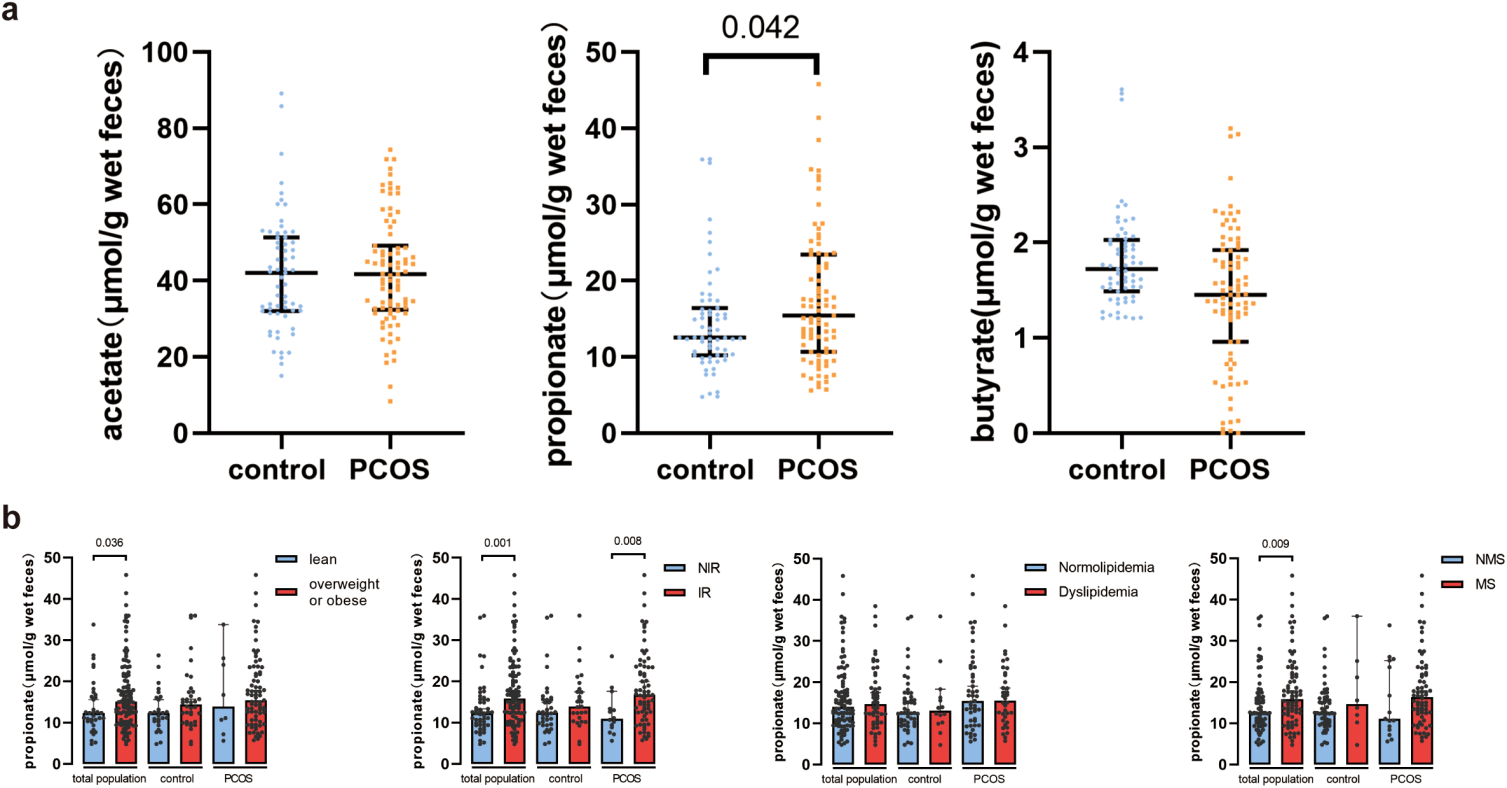
Fecal SCFA levels in healthy controls and PCOS women. **(A)** Comparison of fecal acetate, propionate, and butyrate between healthy controls and PCOS women. Bars represent median with interquartile range. **(B)** Fecal propionate levels between control women and PCOS women in the normal weight and overweight or obese groups, the non-insulin-resistant and insulin-resistant groups, the normolipidemia and dyslipidemia groups, and the non-metabolic syndrome and metabolic syndrome groups. Bars represent the median with interquartile range. PCOS, polycystic ovary syndrome; IR, insulin resistance; MS, metabolic syndrome.

The anthropometric and biochemical characteristics of the participants are detailed in **Table 1**. Women with PCOS showed significantly higher levels of BMI, TC, TG, LDL-C, FPG, FINS, HOMA-IR, FAI, TT, LH, and AMH, while they had lower levels of HDL-C, Prog, PRL, and SHBG. Fecal SCFA levels were quantified, with the results presented in **Figure 2A**. Fecal propionate levels in women with PCOS were significantly higher than those in the control group (*p* = 0.042), while no significant differences were observed for fecal acetate and butyrate between the groups (**Figure 2A**; **Supplementary Table S1**).

**Table 1:** The anthropometric and biochemical characteristics of 73 control and 86 PCOS women.

	Control	PCOS	*p*
*N*	63	83	
Baseline information			
Age (years)	33.00 (30.00–35.00)	31.00 (28.00–33.00)	0.003
BMI (kg/m^2^)	25.08 (21.30–30.18)	29.40 (26.53–32.39)	<0.001
Lipid metabolism			
TC (mM)	4.60 (4.21–5.28)	5.07 (4.40–5.81)	0.014
TG (mM)	0.91 (0.67–1.55)	1.53 (1.12–2.31)	<0.001
HDL-C (mM)	1.43 (1.16–1.65)	1.12 (1.02–1.23)	<0.001
LDL-C (mM)	2.69 (2.45–3.24)	3.24 (2.90–3.75)	<0.001
Glucose metabolism			
FPG (mM)	5.20 (4.95–5.57)	5.42 (5.11–5.90)	0.016
FINS (mIU/L)	9.50 (7.10–13.50)	17.70 (12.30–23.30)	<0.001
HOMA-IR	2.18 (1.49–3.36)	4.44 (2.83–6.08)	<0.001
Sex hormone			
E2 (pg/mL)	36.74 (27.74–49.43)	36.34 (27.56–47.00)	0.494
Prog (ng/mL)	0.54 (0.36–0.78)	0.40 (0.27–0.56)	0.001
PRL (ng/mL)	11.46 (8.71–13.96)	9.10 (6.88–12.12)	0.006
SHBG (nM)	35.80 (24.50–59.80)	18.90 (13.90–26.50)	<0.001
FAI	3.18 (2.30–6.28)	12.97 (8.28–19.36)	<0.001
TT (ng/mL)	0.41 (0.29–0.50)	0.70 (0.58–0.81)	<0.001
LH (mIU/mL)	3.75 (2.85–5.16)	8.89 (7.23–12.32)	<0.001
FSH (mIU/mL)	6.89 (5.67–7.64)	6.41 (5.65–7.59)	0.190
AMH (ng/mL)	3.14 (1.94–4.84)	7.98 (4.55–11.86)	<0.001

PCOS, polycystic ovary syndrome; BMI, body mass index; TC, total cholesterol; TG, triglycerides; HDL-C, high-density lipoprotein cholesterol; LDL-C, low-density lipoprotein cholesterol; FPG, fasting plasma glucose; FINS, fasting insulin; HOMA-IR, homeostasis model assessment of insulin resistance; E2, estradiol; Prog, progesterone; PRL, prolactin; SHBG, sex hormone-binding globulin; FAI, free androgen index; TT, total testosterone; LH, luteinizing hormone; FSH, follicle-stimulating hormone; AMH, anti-Müllerian hormone.

### Fecal propionate levels were higher in PCOS women with insulin resistance than PCOS women without insulin resistance

Considering the role of SCFAs in metabolism, we investigated the relationship between SCFA levels and metabolic indexes. The participants were grouped based on the presence or absence of metabolism disorders, and SCFA levels were compared. Acetate and butyrate levels did not show significant differences between the various groups (**Supplementary Figure S1**). However, fecal propionate levels were significantly higher in the overweight and obese group (*p* = 0.036), the IR group (*p* = 0.001), and the MS group (*p* = 0.009) (**Figure 2B**). We further separated the participants based on their metabolism state and whether they had PCOS. Interestingly, fecal propionate levels were significantly higher in PCOS women with IR compared to PCOS women without IR (*p* = 0.008; **Figure 2B**), but this difference was not observed in the control group with and without IR.

### Fecal propionate levels were correlated with insulin resistance in PCOS women

**Figure 3:**
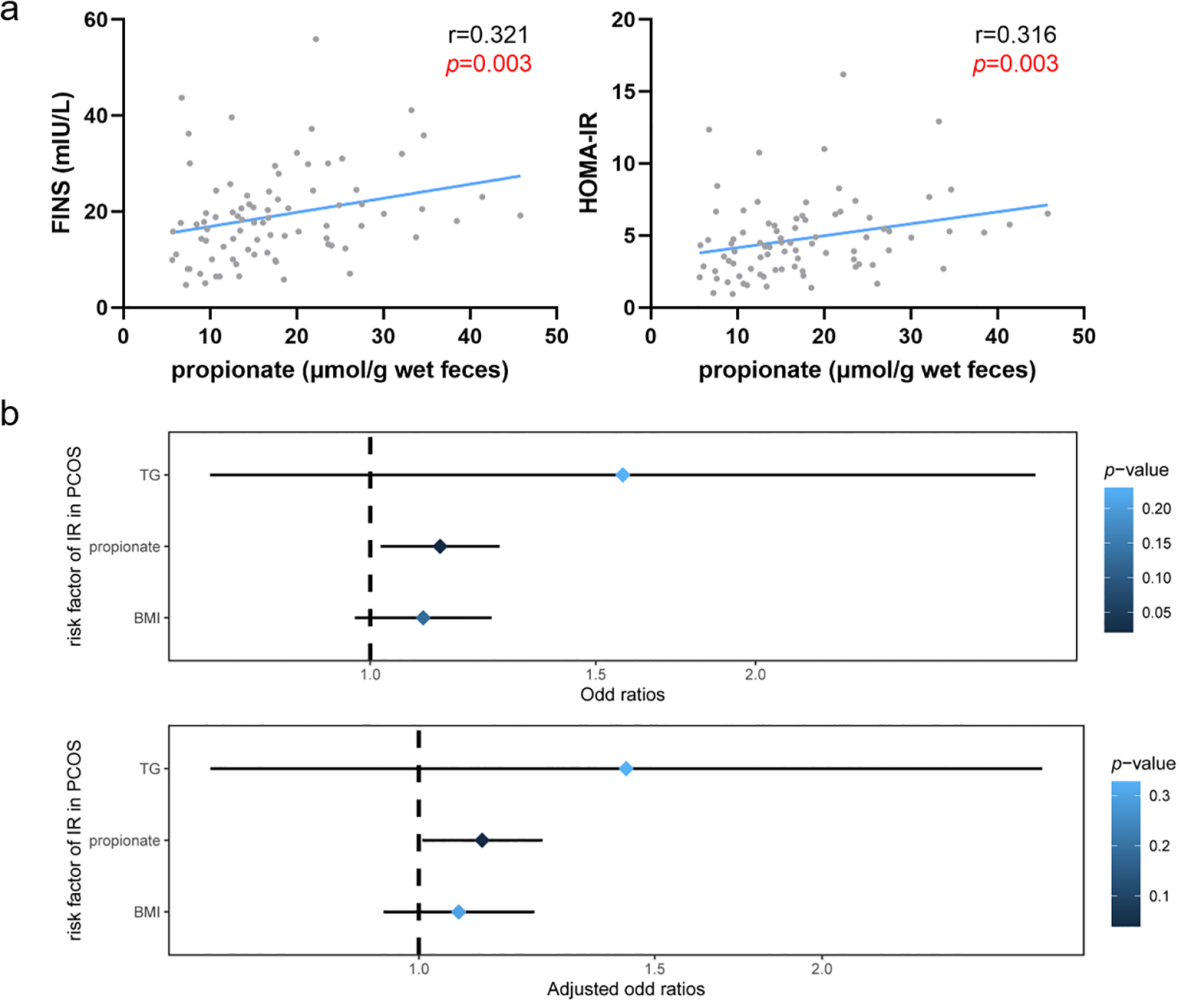
Association between fecal propionate and insulin resistance in PCOS and control women. **(A)** Association between serum FINS and fecal propionate levels in PCOS women and association between HOMA-IR and fecal propionate levels in PCOS women. **(B)** Univariate and multivariate logistic regression of risk factors of IR in PCOS women. FINS, fasting insulin; HOMA-IR, homeostasis model assessment of insulin resistance.

We then explored the relationship between fecal propionate levels and IR. Spearman correlation analyses revealed that fecal propionate levels were positively associated with FINS (*r* = 0.321, *p* = 0.003) and HOMA-IR (*r* = 0.316, *p* = 0.003) in women with PCOS (**Figure 3A**). The characteristics of each participant group categorized by their HOMA-IR are presented in **Table 2**. We used univariate and multivariate linear regression analyses to assess the effects of fecal propionate on FINS in women with PCOS. The results showed that propionate levels were correlated with FINS after adjusting for several covariables, including BMI, SHBG, FPG, TC, TG, and LDL-C levels (*β* coefficient, 0.238; 95% CI, 0.030–0.446; *p* = 0.025; **Table 3**). Logistic regression analysis indicated that fecal propionate increased the risk of IR occurrence (*p* = 0.021; OR, 1.134; 95% CI, 1.019–1.262) and was an independent risk factor for IR after adjusting for BMI and TG, the two indexes that were significantly associated with increased FSI and are known factors related to IR (*p* = 0.039; OR, 1.115; 95% CI, 1.006–1.237) (**Figure 3B**; **Supplementary Table S2**). The interaction terms of propionate, BMI, and TG did not affect the occurrence of IR (*p* = 0.142; OR, 1.003; 95% CI, 0.999–1.006), indicating that no significant interaction effect was observed. Based on the tolerance and variance inflation factor shown in **Supplementary Table S3**, collinearity issues are considered negligible. We also grouped the patients with PCOS based on the tertiles of propionate levels and evaluated the relationship between each tertile and the incidence of IR in these women using logistic regression analysis (**Table 4**). This analysis revealed that the incidence of IR in patients with propionate levels greater than 19.00 μmol/g wet feces was significantly higher (*p* = 0.038; OR, 10.128; 95% CI, 1.135–90.406) compared to those with propionate levels less than 12.63 μmol/g wet feces. Taken together, these findings suggest that higher fecal propionate levels are associated with increased IR in patients with PCOS.

**Table 2:** Comparison of the anthropometric and biochemical characteristics for each group of study participants categorized based on their HOMA-IR.

	Control			PCOS		
	NIR	IR	*p*-value	NIR	IR	*p*-value
***N***	36	27		14	69	
Baseline information						
**Age (years)**	33.00 (31.00–35.00)	33.00 (28.00–35.00)	0.845	30.50 (29.00–35.00)	31.00 (28.00–33.00)	0.660
**BMI (kg/m^2^)**	22.31 (20.47–25.90)	29.03 (25.64–33.25)	<0.001	28.64 (21.47–31.18)	30.07 (26.79–32.95)	0.195
Lipid metabolism						
**TC (mM)**	4.53 (4.20–5.05)	4.61 (4.22–5.52)	0.359	5.39 (4.27–5.83)	4.97 (4.41–5.77)	0.985
**TG (mM)**	0.76 (0.62–1.02)	1.43 (0.91–1.82)	<0.002	1.44 (0.98–2.07)	1.58 (1.15–2.37)	0.337
**HDL-C (mM)**	1.49 (1.28–1.76)	1.25 (0.98–1.44)	0.003	1.16 (1.08–1.44)	1.11 (1.02–1.21)	0.139
**LDL-C (mM)**	2.68 (2.49–3.00)	2.88 (2.41–3.61)	0.449	3.35 (2.22–3.78)	3.22 (2.93–3.70)	0.918
Glucose metabolism						
**FPG (mM)**	5.03 (4.62–5.20)	5.52 (5.32–5.74)	<0.001	5.24 (4.86–5.50)	5.50 (5.12–6.12)	0.044
**FINS (mIU/L)**	7.42 (5.75–8.58)	13.90 (11.70–18.70)	<0.001	7.05 (6.31–9.60)	19.20 (15.00–24.45)	<0.001
Sex hormone						
**E2 (pg/mL)**	40.72 (33.27–56.04)	34.00 (24.13–45.00)	0.078	34.89 (26.21–46.00)	36.54 (27.78–47.00)	0.738
**Prog (ng/mL)**	0.54 (0.38–0.69)	0.54 (0.33–0.80)	0.808	0.47 (0.35–0.74)	0.38 (0.24–0.55)	0.107
**PRL (ng/mL)**	11.48 (8.77–16.29)	11.46 (8.60–13.29)	0.555	9.24 (6.68–12.50)	9.10 (6.89–12.12)	0.971
**SHBG (nM)**	54.21 (34.13–72.78)	28.00 (18.20–35.00)	<0.001	24.40 (13.98–37.73)	18.80 (13.00–25.30)	0.082
**FAI**	2.54 (2.08–3.86)	4.69 (3.18–8.11)	<0.001	8.87 (6.31–17.81)	13.07 (9.01–20.16)	0.155
**TT (ng/mL)**	0.42 (0.30–0.51)	0.37 (0.29–0.49)	0.541	0.70 (0.60–0.77)	0.70 (0.58–0.83)	0.846
**LH (mIU/mL)**	4.16 (3.09–5.08)	3.46 (2.40–5.51)	0.307	9.01 (6.45–11.84)	8.82 (7.24–12.34)	0.976
**FSH (mIU/mL)**	7.15 (6.06–8.34)	6.55 (5.31–7.23)	0.053	6.29 (5.43–7.63)	6.41 (5.65–7.56)	0.756
**AMH (ng/mL)**	3.21 (1.97–4.83)	3.14 (1.94–4.86)	0.890	10.09 (7.00–13.77)	7.60 (4.30–11.85)	0.078
SCFAs						
**Acetate (μmol/g wet feces)**	40.70 (31.61–50.96)	42.01 (32.06–52.91)	0.657	34.37 (30.63–41.48)	44.17 (32.67–54.87)	0.089
**Propionate (μmol/g wet feces)**	12.43 (9.67–15.66)	13.93 (10.35–18.17)	0.201	**10.89 (8.54**–**14.42)**	**16.72 (12.41**–**23.61)**	**0.009**
**Butyrate (μmol/g wet feces)**	10.16 (6.93–14.07)	8.74 (5.61–16.30)	0.945	9.80 (8.74–16.07)	10.48 (7.16–15.61)	0.789
Dietary habit						
**Vegetarian-based diet (*n*)**	2	3	0.325	1	2	0.640
**Meat-based diet (*n*)**	4	6		1	9	
**Meat and vegetarian diet (*n*)**	30	18		12	58	

PCOS, polycystic ovary syndrome; BMI, body mass index; TC, total cholesterol; TG, triglycerides; HDL-C, high-density lipoprotein cholesterol; LDL-C, low-density lipoprotein cholesterol; FPG, fasting plasma glucose; FINS, fasting insulin; E2, estradiol; Prog, progesterone; PRL, prolactin; SHBG, sex hormone-binding globulin; FAI, free androgen index; TT, total testosterone; LH, luteinizing hormone; FSH, follicle-stimulating hormone; AMH, anti-Müllerian hormone. The bold values represent statistically significant results (p < 0.05), highlighting key data points.

**Table 3:** Linear regression describing the relationship between fecal propionate and FSI levels in patients with PCOS.

	Univariate regression			Multivariate regression		
	*β* coefficient	95% CI	*p*-value	*β* coefficient	95% CI	*p*-value
**BMI (kg/m^2^)**	0.583	0.165 to 1.000	0.007	0.407	0.013 to 0.800	0.043
**SHBG (nM)**	−0.036	−0.158 to 0.086	0.557			
**TT (ng/mL)**	0.411	−7.980 to 8.802	0.923			
**FPG (mM)**	3.770	0.788 to 6.753	0.014	1.957	−0.823 to 4.737	0.165
**TC (mM)**	0.371	−1.915 to 2.657	0.748			
**TG (mM)**	1.965	1.034 to 2.897	<0.001	1.922	1.062 to 2.782	<0.001
**LDL-C (mM)**	−1.759	−4.280 to 0.762	0.169			
**Propionate (μmol/g wet feces)**	**0.294**	**0.058 to 0.530**	**0.015**	**0.238**	**0.030 to 0.446**	**0.025**

PCOS, polycystic ovary syndrome; CI, confidence interval; BMI, body mass index; SHBG, sex hormone-binding globulin; TT, total testosterone; FPG, fasting plasma glucose; TC, total cholesterol; TG, triglycerides; LDL-C, low-density lipoprotein cholesterol. The bold values represent statistically significant results (p < 0.05), highlighting key data points.

**Table 4:** Logistic analysis of the correlation between propionate level (tertiles) and risk of insulin resistance in women with PCOS.

Propionate (μmol/g wet feces)	OR	95% CI	*p*-value	Adjusted OR^a^	95% CI	*p*-value
**<12.63**	1.000	–	–	1.000	–	–
**12.63–19.00**	2.133	0.597–7.624	0.244	1.785	0.475–6.711	0.391
**≥19.00**	**12.000**	**1.380–104.336**	**0.024**	**10.128**	**1.135–90.406**	**0.038**

PCOS, polycystic ovary syndrome; OR, odds ratio; CI, confidence interval. The bold values represent statistically significant results (p < 0.05), highlighting key data points.

a OR was adjusted for age, BMI, and TG.

### Difference in species of bacteria between PCOS-IR and PCOS-NIR groups

**Figure 4:**
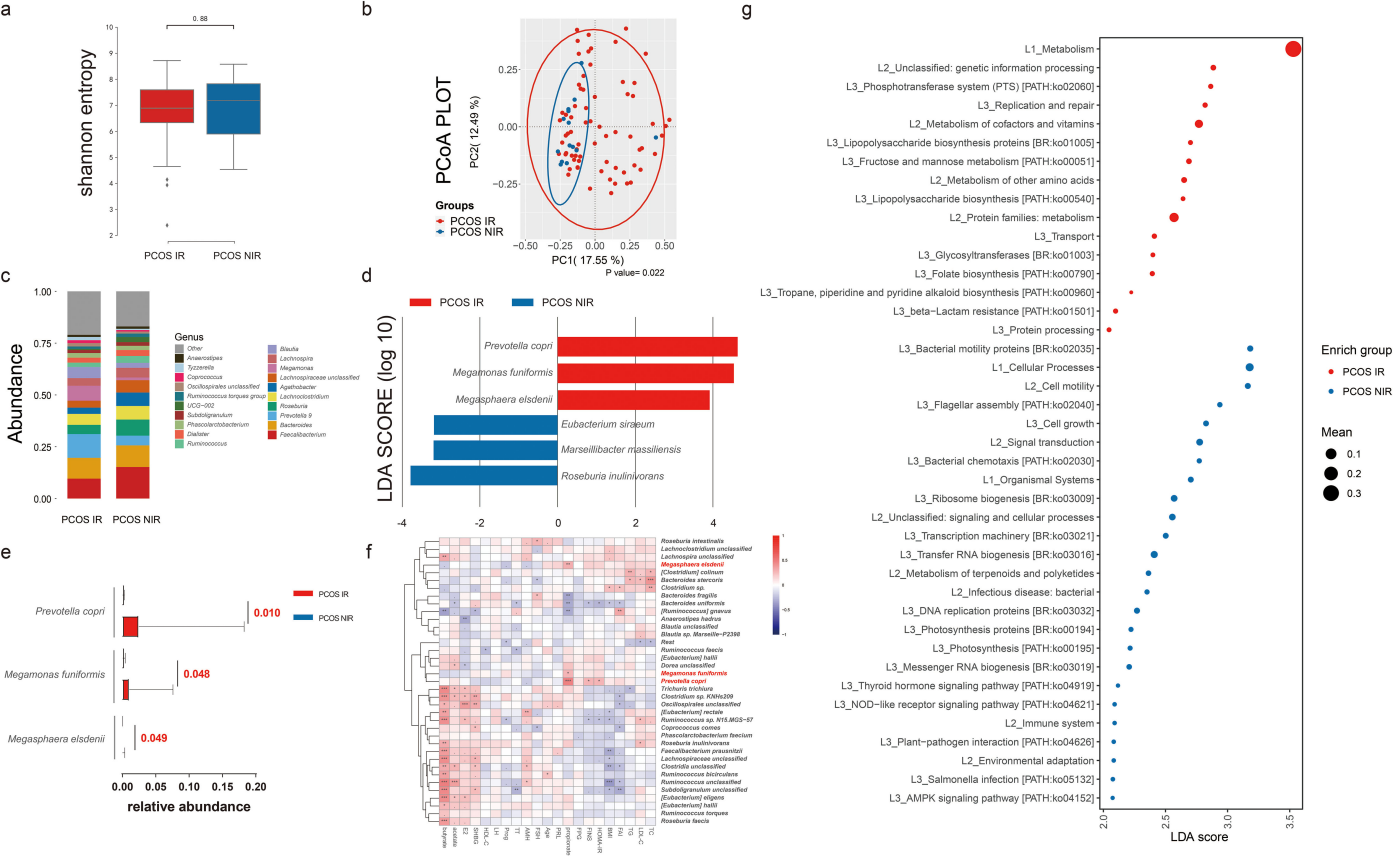
Intestinal microbiome analysis of PCOS women with or without insulin resistance. **(A)** Alpha diversity comparison between the PCOS-IR and PCOS-NIR groups. **(B)** Beta diversity analysis conducted by principal coordinates analysis (PCoA) between the PCOS-IR and PCOS-NIR groups. **(C)** Percentage of bacterial community abundance at the genus level; genera with a relative abundance <1% in each sample are merged into others. Box plots show median ± quartiles, and the whiskers extend from the hinge to the largest or smallest value no further than 1.5-fold of the interquartile range. **(D)** Linear discriminant analysis effect size (LEfSe) for species with different abundances in the PCOS-IR and PCOS-NIR groups. **(E)** Comparison of *Prevotella copri*, *Megamonas funiformis*, and *Megasphaera elsdenii* abundance between the PCOS-IR group and the PCOS-NIR group. **(F)** Correlation between top species in PCOS and clinical indexes and fecal SCFA levels in PCOS women. PCOS, polycystic ovarian syndrome; IR, insulin resistance; NIR, non-insulin resistance; BMI, body mass index; TC, total cholesterol; TG, triglycerides; HDL-C, high-density lipoprotein cholesterol; LDL-C, low-density lipoprotein cholesterol; FPG, fasting plasma glucose; FINS, fasting insulin; E2, estradiol; Prog, progesterone; PRL, prolactin; SHBG, sex hormone-binding globulin; FAI, free androgen index; TT, total testosterone; LH, luteinizing hormone; FSH, follicle-stimulating hormone; AMH, anti-Müllerian hormone. **(G)** Difference analysis based on functions in KEGG pathways between IR and NIR in PCOS women. *P < 0.05, **P < 0.01, ***P < 0.001.

We then set out to explore the bacteria characteristic of PCOS-IR versus PCOS-NIR. Samples with more than 5,000 contigs were filtered after sequencing full-length 16S rRNA, and all samples met the standard. We assessed α diversity using the Shannon index based on OTUs with QIIME 2. We observed a decreased Shannon index in PCOS women (*p* = 0.08, **Figure 4A**), indicating a decrease in α diversity in the PCOS-IR group when compared to the PCOS-NIR group. In the control group, there was almost no difference in α diversity between the IR and NIR groups (**Supplementary Figure S2A**). Beta diversity based on ASVs was assessed by PCoA. The results did not separate the PCOS and control groups (**Figure 4B**). The microbial composition at the phylum and genus levels is shown in **Figure 4C**. *Prevotella copri*, *Megamonas funiformis*, and *Megasphaera elsdenii* were more abundant in PCOS-IR women than in PCOS-NIR women, while *Roseburia inulinivorans*, *Marseillibacter massiliensis*, and *Eubacterium siraeum* were more abundant in PCOS-NIR women than PCOS-IR women, each with an LDA score over 3 (**Figure 4D**). The abundance of *P. copri*, *M. funiformis*, and *M. elsdenii* in the PCOS-IR group was significantly higher than in the PCOS-NIR group (**Figure 4E**). Moreover, these three species were positively correlated with fecal propionate levels (*p* < 0.001), as well as FINS and HOMA-IR (*p* < 0.05, **Figure 4F**). In the control group, only *E. siraeum* was more abundant in control NIR women than in the control IR group, with an LDA score >3 (**Supplementary Figure S2D**). The functional differences in the microbiome between the PCOS-IR and PCOS-NIR groups, based on the KEGG pathways, are shown in **Figure 4G**. The phosphotransferase system was highly enriched in the microbiota of the PCOS-IR group. Additionally, pathways related to carbohydrate metabolism, specifically fructose and mannose metabolism, was also enriched in the PCOS-IR population. Furthermore, the abundance of genes related to lipopolysaccharide (LPS) biosynthesis was higher in the PCOS-IR group. These findings suggested that the microbiota of individuals with PCOS-IR may have a higher capacity for carbohydrate utilization and elevated levels of proinflammatory factors. However, there were no significant functional differences in metabolism between the IR and NIR groups within the control group (**Supplementary Figure S2E**). We also compared the intestinal microbiome structures between the different dietary subgroups. The results are shown in **Supplementary Figure S3**. The results show that bacteria such as *Bacteroides* were characteristic bacteria in the meat-based diet group, while *Lactobacillus* and other bacteria were characteristic bacteria in the vegetarian-based diet group, suggesting that in this study, dietary structure is not the primary factor influencing IR in women with PCOS.

### Prediction performance for IR in PCOS of fecal propionate and related bacteria

**Figure 5:**
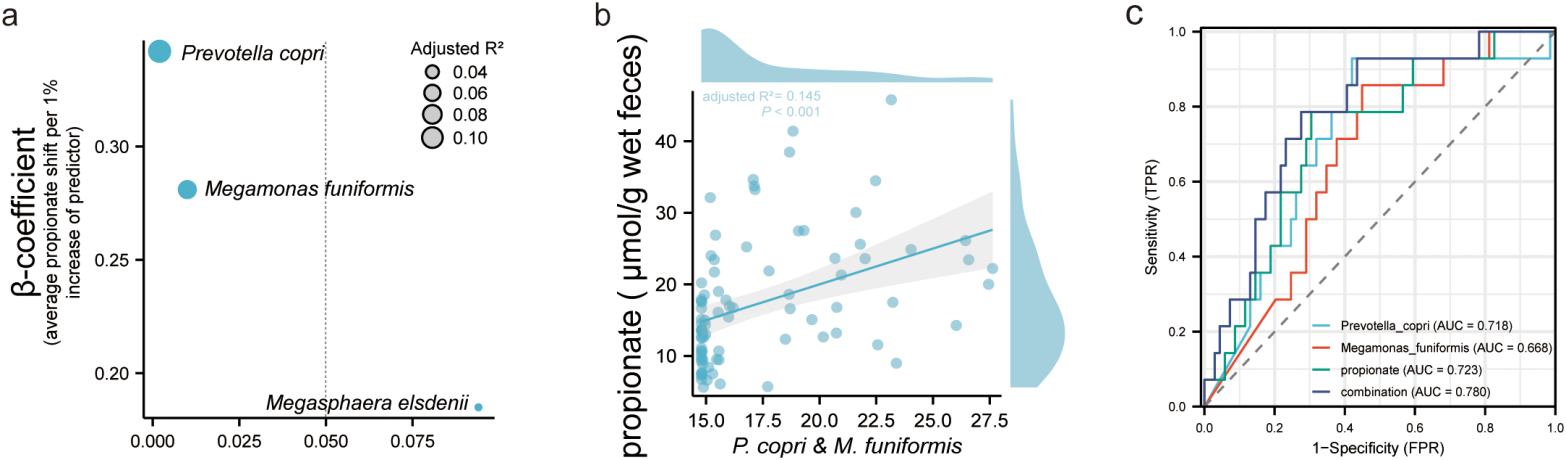
Fecal propionate level in PCOS women could be explained by intestinal microbiota. **(A)** Univariate linear regression analysis determining the characteristic bacteria that predict the fecal propionate. The *y*-axis shows the *β* coefficient for each predictor, as in the average fecal propionate level when their relative abundance increases 1%. The *x*-axis shows the *p*-value for each predictor. Bubble size represents the adjusted *R* ^2^. **(B)** Scatter plots show the linear relationship between fecal propionate level and the contribution of *Prevotella copri* and *Megamonas funiformis*. The shaded area specifies the 95% confidence interval. **(C)** ROC curves illustrate the value of fecal propionate, *Prevotella copri*, and *Megamonas funiformis* in predicting IR in PCOS women. PCOS, polycystic ovary syndrome; ROC, receiver operating characteristic; AUC, the area under the curve.

Univariate linear regression analysis was applied to determine the connections between characteristic bacteria and fecal propionate levels in women with PCOS (**Figure 5A**). This analysis revealed that *P. copri* (adjusted *R^2^* = 0.106, *p* = 0.002) and *M. funiformis* (adjusted *R^2^* = 0.067, *p* = 0.010) but not *M. elsdenii* showed a stronger linear relationship with fecal propionate levels. Multivariate linear relationships between fecal propionate and significant predictors were further visualized using scatter plots (**Figure 5B**), reaffirming the correlations (adjusted *R^2^* = 0.145, *p* < 0.001).

Finally, we explored the clinical value of fecal propionate levels and the abundance of its related bacteria in women with PCOS. We assessed the prediction performance using ROC curves. The AUC of fecal propionate for predicting IR in women with PCOS was 72.3%, with a sensitivity of 78.5% and specificity of 69.5% at a cutoff value of 13.403 μmol/g wet feces. The AUCs of *P. copri* and *M. funiformis* were 71.8% and 66.8%, respectively (**Figure 5C**; **Supplementary Table S4**). We further explored the performance of combining fecal propionate levels with the abundance of these two bacteria. This combination yielded an AUC of 78.0%, with a sensitivity of 78.5% and specificity of 72.4% for predicting IR in women with PCOS (**Figure 5C**; **Supplementary Table S3**).

## Discussion

Mounting evidence supports the critical role of fecal SCFAs in the reproductive system (Olaniyi et al., 2022). This study demonstrated that fecal propionate levels were significantly higher in women with PCOS and were associated with IR in PCOS women after adjustment for covariates. Similarly, Li’s study also showed that fecal propionate levels in women with PCOS were higher than those in the control group (Li et al., 2022). However, Zhang’s study reported that fecal propionate levels in women with PCOS were lower than those in the control group (Zhang et al., 2019). SCFA levels are closely related to diet (Louis and Flint, 2017), and we speculate that differences in dietary habits and gut microbiota among different populations may have led to these inconsistent results. In our study, the number of PCOS-IR women with a meat-based diet was higher than those in the PCOS-NIR group. Some studies have shown that individuals with relatively high *Prevotella* abundance lose more weight on diets rich in dietary fiber (Christensen et al., 2019; Christensen et al., 2020; Hjorth et al., 2020). In our study, the abundance of *P. copri* in women in the PCOS-IR group was significantly higher than in PCOS-NIR women. We speculate that these factors might promote metabolic abnormalities, yet further studies are needed to elucidate the underlying mechanism.

Metabolic dysfunction characterized by IR is a key element contributing to the progression of PCOS (Azziz, 2018). Several studies have reported conflicting results on the association between propionate intake and IR. Tirosh’s study demonstrated that propionate leads to IR and hyperinsulinemia (Tirosh et al., 2019). Conversely, other studies found that oral or rectal supplementation of propionate had no effect on blood glucose or insulin levels (Laurent et al., 1995) or even reduced them (Venter et al., 1990; Todesco et al., 1991). Our results indicated that fecal propionate levels in PCOS women with IR were higher than those in PCOS women without IR, and this difference in fecal propionate levels seemed unique to women with PCOS. Additionally, fecal propionate concentrations were positively correlated with both the FINS level and HOMA-IR in PCOS. These findings suggest that IR may be a key link between propionate and PCOS. Although the exact correlation between propionate and IR, as well as the underlying mechanisms of these interactions, remains elusive, these initial results shed light on the potential role of propionate in PCOS-related insulin metabolism disorders.

We further explored the factors driving this increase of fecal propionate levels in the PCOS-IR group. Our results indicated that fecal propionate could be predicted by intestinal microbiota, specifically *P. copri* and *M. funiformis*. *Prevotella copri* encodes metabolic pathways for propionate production (Louis and Flint, 2017). *Megamonas genus* also contributes to propionate production (Polansky et al., 2015). Additionally, *M. funiformis* is associated with systemic lupus erythematosus, T2DM, and other diseases (Balmant et al., 2023; Zhang et al., 2023). Our findings showed that the abundances of *P. copri* and *M. funiformis* were increased in the PCOS-IR population and were significantly correlated with fecal propionate levels. We speculate that these bacteria may promote the occurrence and development of IR in PCOS patients through propionate production. However, the specific mechanisms require further experimental research.

The major strength of this study lies in being the first to assess the fecal propionate levels in women with PCOS and evaluate their relationships with both PCOS and PCOS-related IR. We found that propionate levels differed significantly between IR and NIR individuals only in the PCOS group, but not in the control group. This suggests that PCOS symptoms may create a specific correlation between propionate and IR, warranting further investigation into the underlying mechanisms. Additionally, our findings have clinical implications: fecal propionate levels, combined with relevant gut microbiota, could serve as a biomarker for predicting IR in women with PCOS. This could be crucial for managing PCOS in clinical settings. Our results underscore the potential clinical value of measuring fecal propionate levels in daily practice.

Nevertheless, our study has several limitations. First, it was a single-center study, and future research should validate these findings through multicenter studies. Second, the study was small and cross-sectional, so our data should be interpreted with caution and within the context of this design. Third, PCOS presents with heterogeneous phenotypes, and we were unable to separately evaluate the impact of each phenotype on fecal propionate levels, which may limit the generalizability of our results.

Overall, the current study highlights that higher fecal propionate concentrations are positively correlated with PCOS-related IR and may serve as a potential predictive biomarker for IR in PCOS. Further prospective and experimental studies are needed to confirm these findings and to elucidate the molecular mechanisms underlying IR in the development and progression of PCOS.

## Data Availability

The 16S dataset generated and analyzed during the current study, numbered PRJNA1094483, is available on SRA datasets.
