# Supplementary material for: Fecal propionate is a signature of insulin resistance in polycystic ovary syndrome
Source: Front Cell Infect Microbiol. 2025 Jan 13;14:1394873. doi: 10.3389/fcimb.2024.1394873 (PMC11769941; doi:10.3389/fcimb.2024.1394873)
Supplement: Supplementary file 1 [file Table1.docx]

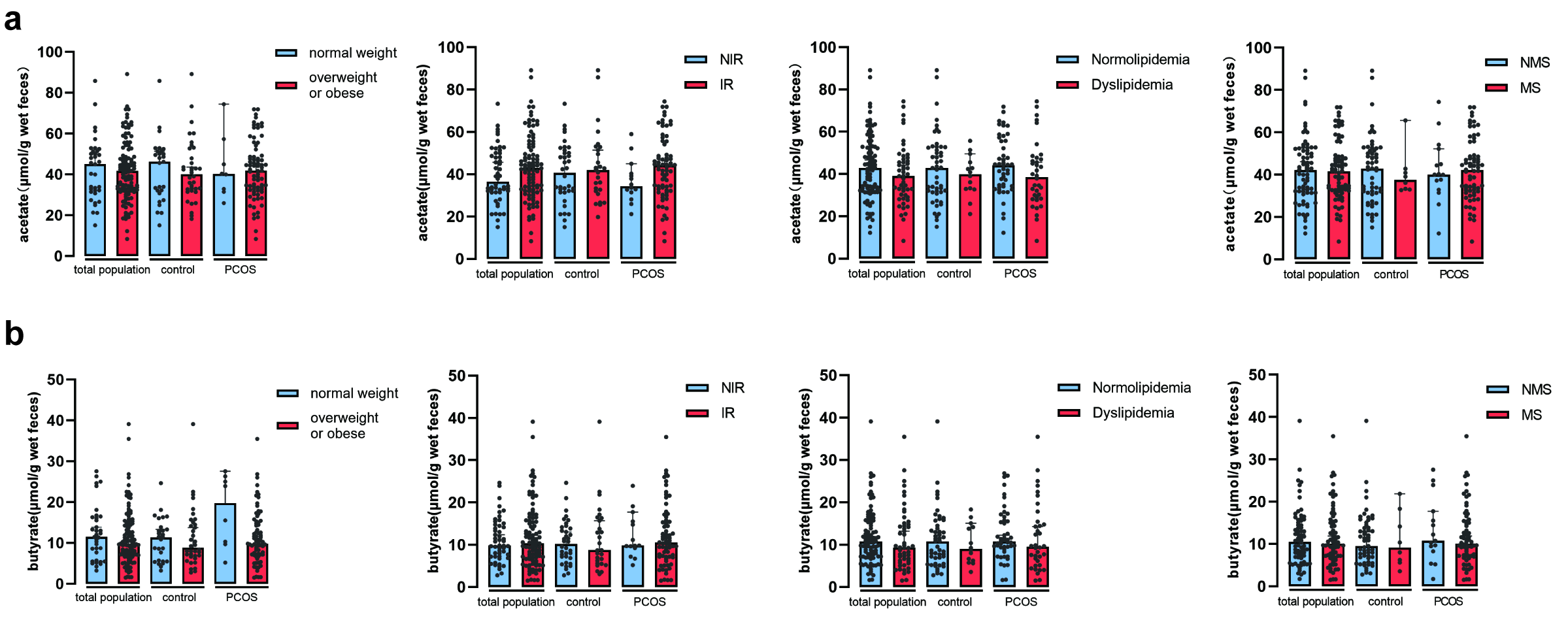


Supplementary figure 1 *Fecal acetate and butyrate levels in healthy controls and PCOS women.* Differences in fecal a, acetate; b, butyrate levels between control women and PCOS women in the normal weight and overweight or obese groups, the noninsulin-resistant and insulin-resistant groups, the normolipidemia and dyslipidemia groups, the nonmetabolic syndrome and metabolic syndrome groups. Bars represents the median with interquartile range. PCOS, polycystic ovary syndrome; IR, insulin resistance; MS, metabolic syndrome.





Supplementary figure 2 *Intestinal microbiome analysis of healthy control women with or without insulin resistance.* a, Alpha diversity comparison between control IR and control NIR groups. b, Beta diversity analysis conducted by principal co-ordinates analysis (PCoA) between control IR and control NIR groups. c, Percentage of bacterial community abundance at genus level; genera with a relative abundance <1% in each sample are merged into others. Box plots show median ± quartiles, and the whiskers extend from the hinge to the largest or smallest value no further than 1.5 folds of the inter-quartile range. d, Linear discriminant analysis effect size (LEfSe) for species with different abundances in control IR and control NIR groups. e, Difference analysis based on functions in KEGG and pathways between IR and NIR in control group.


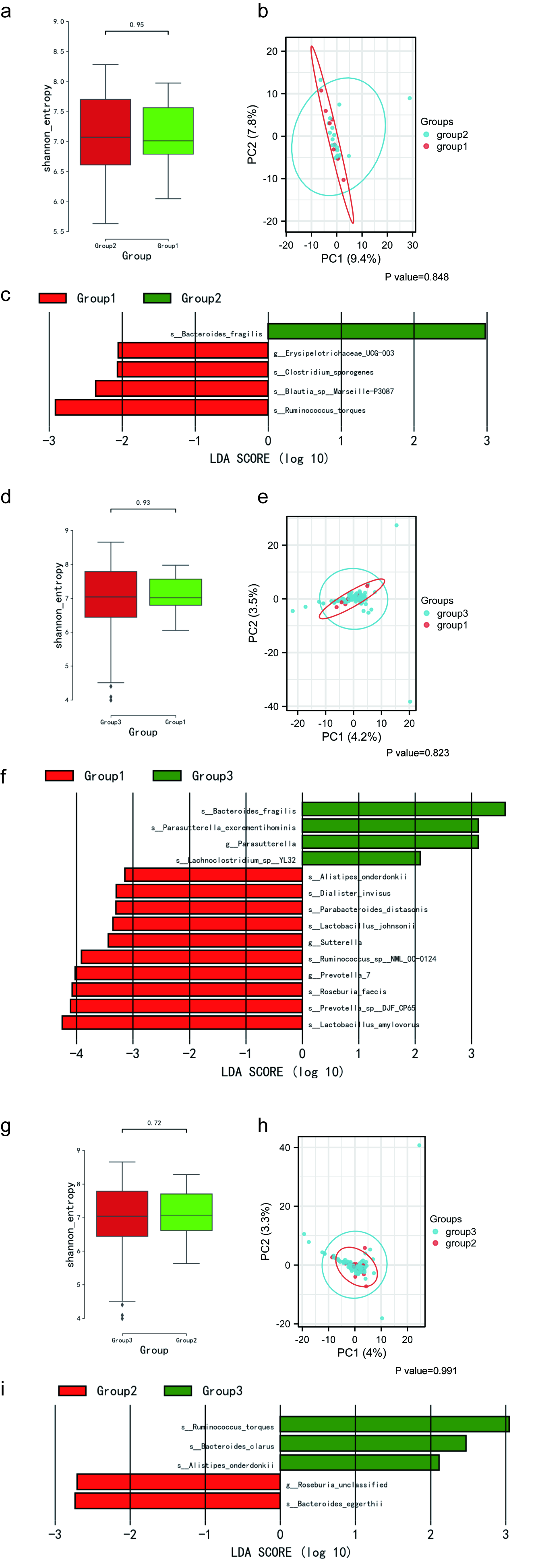


Supplementary figure 3 *Composition of the gut microbiome between different dietary subgroups.*a, Alpha diversity comparison between vegetarian based diet (group1) and meat based diet (group2). b, Beta diversity analysis conducted by principal co-ordinates analysis (PCoA) between group1 and group2. c, Linear discriminant analysis effect size (LEfSe) for species with different abundances in group1 and group2. d, Alpha diversity comparison between group1 and meat and vegetarian diet (group3). e, Beta diversity analysis conducted by principal co-ordinates analysis (PCoA) between group1 and group3. f, Linear discriminant analysis effect size (LEfSe) for species with different abundances in group1 and group3. g, Alpha diversity comparison between group2 and group3. h, Beta diversity analysis conducted by principal co-ordinates analysis (PCoA) between group2 and group3. i, Linear discriminant analysis effect size (LEfSe) for species with different abundances in group2 and group3.
