# Supplementary material for: Fecal propionate is a signature of insulin resistance in polycystic ovary syndrome
Source: Front Cell Infect Microbiol. 2025 Jan 13;14:1394873. doi: 10.3389/fcimb.2024.1394873 (PMC11769941; doi:10.3389/fcimb.2024.1394873)
Supplement: Supplementary file 2 [file Table2.docx]

Supplementary table 1 Levels of fecal acetate, propionate and butyrate in both groups. PCOS, polycystic ovarian syndrome.

| **SCFAs**  **(μmol/g wet feces)** | **Control** | **PCOS** | ***p*-value** |
| --- | --- | --- | --- |
| **acetate** | 42.01(32.06-51.34) | 41.67(32.38-49.24) | 0.784 |
| **propionate** | **12.55(10.21-16.43)** | **15.44(10.68-23.43)** | **0.042** |
| **butyrate** | 9.53(6.23-14.48) | 10.29(7.19-15.51) | 0.390 |

Supplementary table 2 Logistic analysis of the correlation between propionate concentration and risk of insulin resistance in women with PCOS.

|  | **Univariate regression** | | | **Multivariate regression** | | |
| --- | --- | --- | --- | --- | --- | --- |
|  | **OR** | **95%CI** | ***p*-value** | **OR** | **95%CI** | ***p*-value** |
| **BMI (kg/m^2^)** | 1.100 | 0.973-1.244 | 0.129 | 1.071 | 0.941-1.220 | 0.299 |
| **TG (mM)** | 1.575 | 0.750-3.307 | 0.230 | 1.428 | 0.699-2.919 | 0.328 |
| **Propionate**  **(μmol/g wet feces)** | **1.134** | **1.019-1.262** | **0.021** | **1.115** | **1.006-1.237** | **0.039** |

**Abbreviations:** PCOS, polycystic ovary syndrome; OR, odds ratio; CI, confidence interval; BMI, body mass index; TG, triglycerides.

Supplementary table 3 Diagnostic criteria for collinearity.

| Coefficient^a^ | | | | | | | |
| --- | --- | --- | --- | --- | --- | --- | --- |
|  | Non standardized coefficient | | Standardized coefficient |  |  | Collinearity | |
|  | B | standard error | Beta | t | P-value | Tolerance | Variance inflation factor |
| Constant | -0.469 | 0.179 |  | -2.625 | 0.010 |  |  |
| BMI (kg/m^2^) | 0.033 | 0.006 | 0.393 | 5.199 | 0.000 | 0.927 | 1.078 |
| TG (mM) | 0.036 | 0.018 | 0.152 | 2.045 | 0.043 | 0.958 | 1.044 |
| Propionate  (μmol/g wet feces) | 0.008 | 0.004 | 0.138 | 1.843 | 0.067 | 0.944 | 1.060 |

1. Dependent variable：HOMA-IR2.5

Supplementary table 4 Prediction performance of fecal propionate levels for insulin resistance in patients with PCOS.

| **Insulin resistance in PCOS** | **AUC** | **Cut off** | **95%CI** | **Sensitivity%** | **Specificity%** |
| --- | --- | --- | --- | --- | --- |
| ***Prevotella copri*** | 0.718 | 0.007 | 0.575 - 0.861 | 92.8 | 57.9 |
| ***Megamonas funiformis*** | 0.668 | 0.005 | 0.531 - 0.804 | 85.7 | 55.0 |
| **Fecal propionate** | 0.723 | 13.403 | 0.583 - 0.862 | 78.5 | 69.5 |
| **Combination** | 0.780 | 0.205 | 0.654 - 0.905 | 78.5 | 72.4 |

PCOS, polycystic ovary syndrome; ROC, receiver operating characteristic; AUC, the area under the curve; SE, standard error; CI, confidence interval.
